# The Bat Signal: An Ultraviolet Light Lure to Increase Acoustic Detection of Bats

**DOI:** 10.3390/ani15162458

**Published:** 2025-08-21

**Authors:** Samuel R. Freeze, Sabrina M. Deeley, Amber S. Litterer, J. Mark Freeze, W. Mark Ford

**Affiliations:** 1 Department of Fish and Wildlife Conservation, Virginia Polytechnic Institute and State University, Blacksburg, VA 24061, USA; amberlitterer@vt.edu; 2 Conservation Planning Assistance, Chesapeake Bay Ecological Services Field Office, U.S. Fish and Wildlife Service, Annapolis, MD 21401, USA; sabrina_deeley@fws.gov; 3 Independent Electrical Engineer, Raleigh, NC 27613, USA; jmfreeze2@gmail.com; 4 Virginia Cooperative Fish and Wildlife Research Unit, U.S. Geological Survey, Blacksburg, VA 24061, USA; wmford@vt.edu

**Keywords:** acoustic survey, bats, detection, echolocation, endangered species, feeding buzz, lure, ultraviolet light

## Abstract

Bats are a taxa of high conservation concern and are facing numerous threats including widespread mortality due to White-Nose Syndrome (WNS) in North America. With this decline comes increasing difficulty in monitoring imperiled bat species due to lower detection probabilities of both mist-netting and acoustic surveys. Lure technology shows promise to increase detection while decreasing sampling effort; however, to date research has primarily focused on increasing physical captures during mist-net surveys using sound lures. Because much bat monitoring is now performed using acoustic detection, there is a similar need to increase detection probabilities during acoustic surveys. Ultraviolet (UV) lights anecdotally have been shown to attract insects and thereby attract foraging bats for observational studies and to experimentally provide a food source for WNS-impacted bats before and after hibernation. Therefore, we constructed a field-portable and programmable UV lure device to determine the value of lures for increasing acoustic detection of bats. We tested if the lure device increased both the echolocation passes and feeding activity (feeding buzzes) across a transect of bat detectors. There was an increase in feeding activity around the UV light, with a nuanced, species-specific and positionally dependent effect on echolocation passes received. The UV light lure increased echolocation passes for the eastern red bat (*Lasiurus borealis*), little brown bat (*Myotis lucifugus*), and evening bat (*Nycticeius humeralis*), but decreased passes of the North American hoary bat (*Lasiurus cinereus*). The northern long-eared bat (*Myotis septentrionalis*) showed a negative response within the illuminated area but increased echolocation activity outside the illuminated area during lure treatment and activity was elevated at all positions after the lure was deactivated. Our study demonstrates some potential utility of UV lures in increasing the feeding activity and acoustic detection of bats. Additional research and development of UV lure technology may be beneficial, including alternating on and off periods to improve detection of light-averse species, and improving echolocation call quality along with the increase in received passes.

## Simple Summary

Scientists often use acoustic recording devices to record ultrasonic (above human hearing) echolocation calls that bats use for navigation and foraging at night. These calls can be identified to bat species for use in environmental studies using automated computer programs. However, due to the rapid attenuation of high-frequency echolocation pulses bats must fly close to the microphone in order to record a clear sound file that can be identified by the software. We built and tested a device that uses ultraviolet (UV) “blacklights” to attract insects thereby potentially attracting foraging bats to an array of recording devices. The lure device worked for individual species of bats such as the eastern red bat (*Lasiurus borealis*) but not the entire bat community. Our primary species of interest, the endangered northern long-eared bat (*Myotis septentrionalis*) was repelled within the area illuminated by the UV light lure but activity increased outside the illuminated area. Overall, a UV light lure shows promise for increasing detection of some species, but caution is recommended because some bats seemed to avoid the light.

## 1. Introduction

Bats are a taxon of high conservation concern globally and are facing numerous stressors and threats [1]. Data necessary to lead conservation and management efforts often are lacking, as bats generally are difficult to study due to their cryptic, nocturnal, and volant life history [2]. This problem has been exacerbated for North American cave-hibernating species by the introduction of White-Nose Syndrome, an infection caused by the fungus *Pseudogymnoascus destructans*, that has resulted in precipitous declines of several species [3]. Declines in mist-netting capture have caused a shift to a greater use of acoustic sampling to study and monitor bats [4,5]. Acoustic sampling has the advantage of allowing researchers to passively survey bats over larger areas and longer durations with less effort than traditional mist-net surveys [6]. Nevertheless, for species such as the endangered northern long-eared bat (*Myotis septentrionalis*) in the eastern United States, acoustic detection probabilities continue to decline concomitantly with continued population reduction [7]. In the mid-Atlantic region of the United States, Deeley et al. [8] showed that more sampling nights and/or sites than is logistically feasible for managers to complete are required to confirm the presence of WNS-impacted species within an acceptable level of statistical error. 

Critical to using acoustic sampling to draw inferences about bats is the collection of numerous, long-duration echolocation search-phase passes that ensure more precise automated bat call identification [9]. Proper placement of bat detector microphones is essential [10], but in some passive detector deployments, bats will pass by the microphone quickly, thereby resulting in short-sequence, unidentifiable echolocation passes being recorded. Moreover, high-frequency echolocation signals, such as those produced by members of the *Myotis* genus, have a relatively short range due to rapid attenuation and geometric spreading in the atmosphere, which degrades the recorded signal [9]. Acoustic surveys are particularly biased against species such as the northern long-eared bat that use high-frequency, short-duration, and low-amplitude echolocation calls [11]. If bats could be drawn closer to the microphone, it would result in recording higher quality call sequences and increasing the detection of low-amplitude bat species. Clearly, there is a compelling need for new field methods to increase the detection of bats for research and clearance surveys, particularly for rare bat species that are of the greatest conservation need.

There is ongoing development of ultrasonic acoustic lures that playback recorded or synthesized bat echolocation, social, and/or feeding buzz calls to improve mist-net and harp trap survey detection probability. The results indicate promise in acoustic lure technology, but studies often demonstrate mixed results and species-specific responses [12,13,14,15]. Additionally, the range of acoustic lures is limited due to the rapid attenuation of ultrasound, and there are inherent limitations in the ability of acoustic lure device speakers to accurately reproduce bat calls across the full range of echolocation frequencies. We would expect the same limitations and mixed results using an acoustic lure during acoustic surveys for bats with the added complication of an acoustic lure generating interference with bat detectors. 

One potential lure alternative is to use ultraviolet lights to create an insect swarm around a bat detector or array of bat detectors and thereby attract foraging insectivorous bats [16,17]. An ultraviolet light lure could potentially increase the number and quality of bat passes recorded, as well as reduce the sampling effort required to detect rare or low amplitude bat species. For example, some species of aerial hawking bats are commonly observed hunting insects near streetlights, so it is well established that some bats take advantage of insect aggregations at light sources [18,19,20]. Smaller, clutter-adapted gleaning bats generally avoid areas illuminated with bright white lights [19,20,21,22,23,24,25]. However, long wave ultraviolet “blacklights” (UV-A, ~320 nm–400 nm; hereafter referred to as UV lights) are used by entomologists to attract nocturnal insects [26,27,28]. Many of these insect species are known prey items for insectivorous bats [17,19]. Because UV lights emit minimal visible light, they may be better suited to attracting both aerial hawking bats and/or bat species adverse to white light. Tamsitt [29] noted that bats were attracted to UV light insect traps, and UV lights have been used for observational bat studies [30,31,32,33,34]. Bell [35], using a 15-watt UV fluorescent tube demonstrated an increase in both insect and bat activity across multiple habitat types in the southwestern United States. Most notably, bats appeared to be attracted to the insect resource rather than the light because bat activity was low on cold and windy nights with little insect activity around the light. Adams et al. [16] noted that the use of UV light increased the quantity and quality of bat search-phase echolocation passes, along with greater bat species richness in Australia. Little brown bat (*Myotis lucifugus*) activity along riparian corridors and near hibernacula entrances showed an increase with the use of UV lights [17]. Relative to hibernacula entry sites, increases in feeding buzz echolocation passes have led researchers to hypothesize that concentrating insect resources and increasing local bat activity might improve bat body condition, i.e., less energy expenditures and greater fat deposition, prior to hibernation entry. In contrast, Gorresen [36] observed that flickering UV lights in conjunction with bat deterrent development for use at wind turbine sites in Hawaii to protect the endangered Hawaiian hoary bat (*Lasiurus cinereus semotus*) did not increase bat activity, though results may have been confounded by an overall low and highly dispersed insect resource. 

To determine if UV lights have promise as a tool to enhance acoustic survey methods for bats, i.e., increase echolocation passes recorded and detection probability, we constructed and tested a field-deployable and programmable UV lure in conjunction with an array of bat detectors. Additionally, we also sought to determine if lures increase echolocation passes recorded and if the passes received indicated feeding activity (feeding buzzes). We predicted that the use of a UV lure would increase overall bat echolocation activity as well as feeding activity. We also predicted that edge and open-space foraging bat species, such as the eastern red bat (*Lasiurus borealis*) and big brown bat (*Eptesicus fuscus*), would have the strongest positive response to the lure and that acoustic detectors placed near the UV lure would record more bat passes than detectors located away from the lure.

## 2. Materials and Methods

### 2.1. Study Area

**Figure 1:**
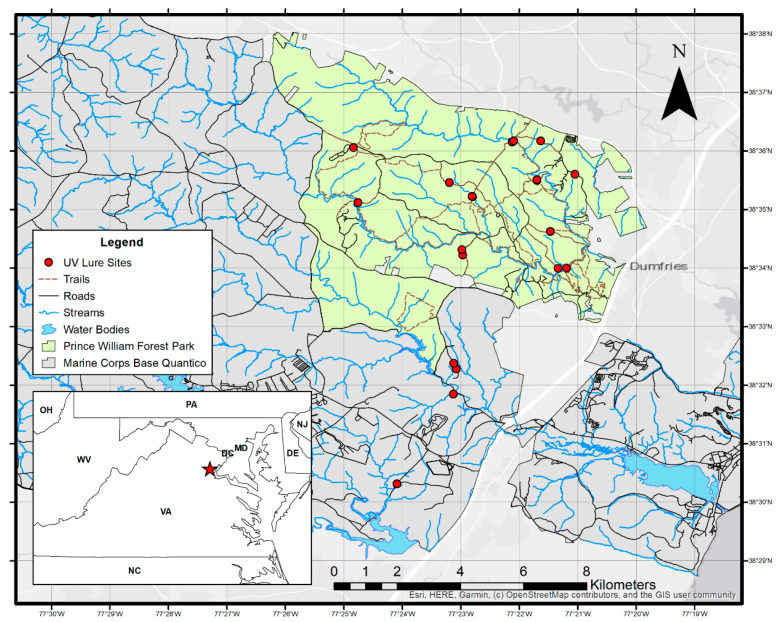
Map of UV light lure sites located in Prince William Forest Park and Marine Corps Base Quantico in eastern Virginia, USA, that were sampled from approximately mid-May to early August, 2020–2021 (https://www.openstreetmap.org/#map=18/38.584564/-77.387250, accessed on 10 October 2021).

Our study was conducted within Prince William Forest Park (PRWI) and the adjacent Marine Corps Base Quantico (MCBQ) in eastern Virginia approximately 50 km south of Washington, D.C. (Figure 1). Prince William Forest Park is a 6070 ha property managed by the National Park Service and is located in the Prince William and Stafford counties. Marine Corps Base Quantico is a 23,876 ha U.S. Department of Defense installation that is adjacent to PRWI to the south and spans the Stafford, Prince William, and Fauquier counties [37,38]. Both PRWI and MCBQ occur along the Fall Line boundary between the mid-Atlantic portions of the northern Piedmont and northern Atlantic Coastal Plain physiographic provinces. The regional climate is “modified continental”, with mild winters and humid summers. Topography in the study area consists of low rolling hills and stream bottomlands with elevations ranging from sea level to 142 meters. Approximately 88% of MCBQ and 95% of PRWI are forested, respectively, with the remainder primarily being open fields, roads right-of-way, water bodies, and developed areas. Most of the forested area is mature second-growth eastern deciduous hardwood forest, typically comprising mixed oak (*Quercus spp.*) with an American beech (*Fagas grandifolia*) understory. Some areas of oldfield-origin Virginia pine (*Pinus virginianus*) and planted loblolly pine (*Pinus taeda*) occur throughout [37,38]. For acoustic recording, we included in the study the bat species that are known or likely to occur in the study area based on previous mist-net captures and acoustic detections in the area combined with range map information. These species included the big brown bat (*Eptesicus fuscus*), eastern red bat (*Lasiurus borealis*), North American hoary bat (*Lasiurus cinereus*; hereafter, hoary bat), silver-haired bat (*Lasionycteris noctivagans*), evening bat (*Nycticius humeralis*), little brown bat (*Myotis lucifugus*), northern long-eared bat (*Myotis septentrionalis*), Indiana bat (*Myotis sodalis*), tricolored bat (*Perimyotis subflavus*), and Brazilian free-tailed bat (*Tadarida brasiliensis*) in the analysis [39].

### 2.2. UV Lure Construction

**Figure 2:**
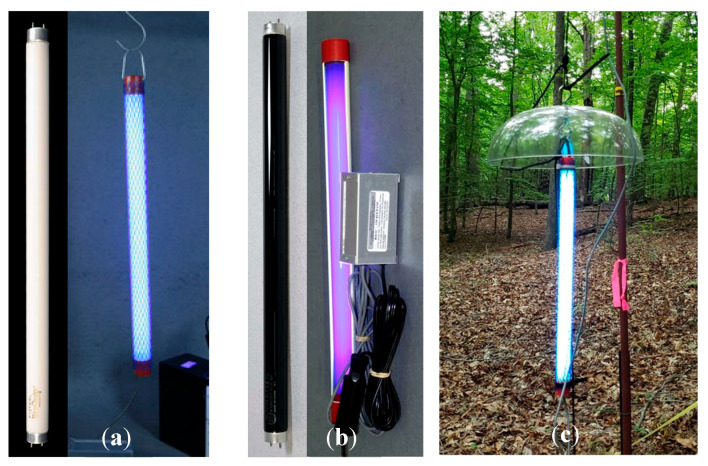
UV lights used in the study at Prince William Forest Park and Marine Corps Base Quantico, VA, USA, 2020, showing: (**a**) an uncoated UV blacklight (BL) fluorescent tube (left) and the tube on demonstrating the brighter output of visible light (right), and (**b**) a coated UV blacklight-blue (BLB) fluorescent tube (left), and the tube on demonstrating less visible light output (right). (**a**,**b**) pictures source: Bioquip, Rancho Dominguez, CA, USA. Accessed: December 2023. (**c**) Close-up picture showing one of the two ultraviolet (UV) fluorescent tubes suspended from a rope that was part of the UV lure used in the study. Note the clear dome is a birdfeeder rain cover used to protect the tube from rain (Samuel Freeze, 2020).

**Figure 3:**
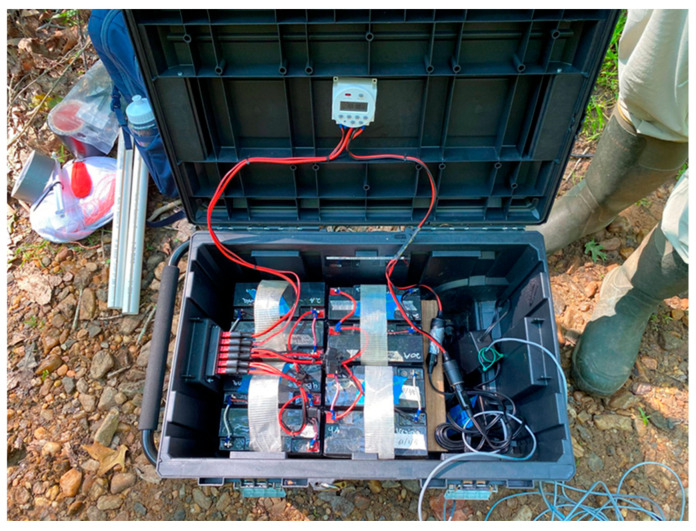
Picture showing the inside of the power supply and control box of the UV lure showing the bank of 12-volt sealed lead-acid batteries connected in parallel via an Anderson power connector distribution block and routed through a programmable timer set to run from sunset to sunrise at Prince William Forest Park and Marine Corps Base Quantico VA, USA, 2020 (Samuel Freeze, 2020).

For the UV lures, we used two 18-inch, 15-watt, 12-volt UV “blacklight” fluorescent tubes that emitted UV light between approximately 320 nm to 400 nm (Bioquip, Model 2805 DC Light, Rancho Dominguez, CA, USA). (Any use of trade, firm, or product names is for descriptive purposes only and does not imply endorsement by the U.S. Government.) We chose to use uncoated standard blacklight (BL) tubes to minimize reductions in UV light emission, though this came at a cost of some emission of visible light [27]. Two basic types of UV fluorescent tubes exist. There are standard blacklight (BL) tubes and coated blacklight-blue (BLB) tubes (Figure 2a,b). Nocturnal insect vision sensitivity peaks in the UV spectrum, but insects are also attracted to green and blue light, which was another factor supporting our use of uncoated BL tubes [27]. Because the UV lights were not weatherproof, we used silicone caulk and electrical tape to seal power connections. We also suspended the lights underneath rain covers (18″ diameter bird feeder rain covers, Aspects Super Dome1, Warren, RI, USA) to further weatherproof the lights (Figure 2c). For a power source, we used eight 12-volt, 18 amp-hour sealed lead acid (SLA) absorbent glass mat (AGM) batteries (Bright Way Group, Dallas, TX, USA; Model BW 12180), taped together in pairs with the leads connected in parallel to facilitate field transport. The four battery pairs were connected in parallel via Anderson power connectors (iGreely1 30A 12v connector, Guangdong, China) with an inline fuse (Camway Trading Limited waterproof fuse holder with 12V 20A fuse, Hong Kong, China) through a distribution block (Chunzehui F-10111 6-Position 45A Power Pole Distribution Block, China). We connected the lights to the power source through a digital programmable timer (FAVOLCANO CN1011, China) that was also connected to the distribution block. The power cables were extended by splicing additional cables onto the existing cable so that the top light had a power cable approximately 6 meters long and the bottom light approximately 3 meters long. The batteries and all electronics were placed in a large, lockable and water-resistant rolling toolbox (Husky 50 gallon1, Atlanta, GA, USA) (Figure 3).

### 2.3. Field Methods

**Figure 4:**
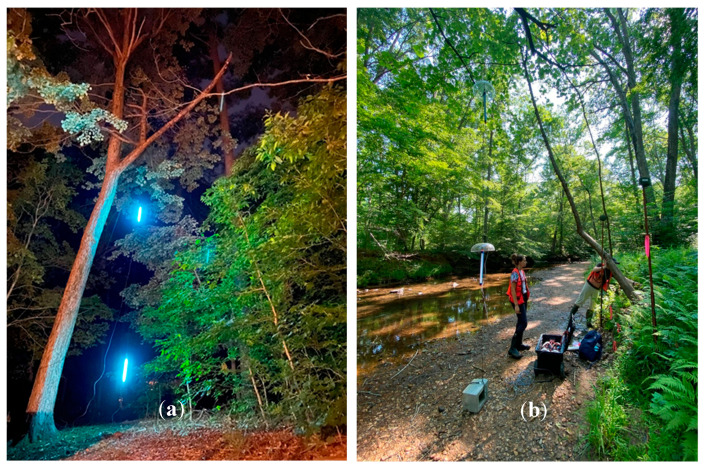
(**a**) Picture showing the UV light lure activated and suspended from a tree in Prince William Forest Park, VA, USA, 2020–2021 (Samuel Freeze, 2020). Note that additional fill light was added to this picture. (**b**) Picture showing a UV lure site deployed along a stream corridor in Prince William Forest Park, VA, USA, 2020–2021 (Samuel Freeze, 2020). Note the two UV lights hanging from the tree with bat detectors on poles located nearby.

**Figure 5:**
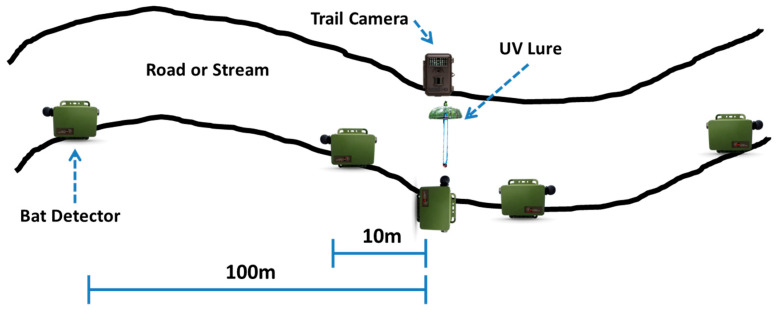
Diagram showing the layout of UV lure sampling site of the five detector positions (center/0 m, 10 m, and 100 m) along with the lure at the center of the site and a trail camera used to confirm lure operation in Prince William Forest Park and Marine Corps Base Quantico, VA, USA, 2020–2021 (Samuel Freeze, 2020).

We conducted UV light trials during the summers of 2020 and 2021 from approximately mid-May to early August to coincide with the summer bat survey season [10]. We placed UV lights along single-track gravel or dirt roads and streams that served as bat flight corridors at PRWI and MCBQ (Figure 4). Survey sites were spaced so that no detector was within 1000 m of another within a given year to reasonably ensure spatial independence during operation. Although research into minimum distances between acoustic recorders to minimize spatial autocorrelation in real time is limited, 30 m was believed sufficient [40]. We chose to be conservative with our minimum distance since we were using a lure device that had potential to attract bats from a larger area. During the summer of 2020, fourteen sites were sampled, and eight sites were sampled during the summer of 2021, with six of those being resampled sites. The UV lure was deployed along the side of the corridor flyway (trail or wooded secondary road) so that there was ample open airspace around the lure for bats to easily forage. We placed Songmeter Mini Bat detectors (Wildlife Acoustics, Maynard, MA, USA) on approximately 2 m tall poles (Mr. LongArm Inc., Greenwood, MO, USA) along the same side of the flyway as the UV lure. One detector was placed at the UV lure and aimed directly at the lights during the summer of 2020, with two during the summer of 2021 to provide redundancy. The horizontal distance between the center detector and UV lure varied slightly between sites but was typically about 1–2 m. To understand the potential effects of distance on UV light placement, we also placed detectors at 10 m and 100 m from the light, with the latter serving as our in-site control (Figure 5). 

We deployed each individual UV light transect for a total of 6 nights, with the first two nights serving as an unlit control, followed by two nights with the UV lure activated and then followed by two more unlit nights to help identify any potential lag effect of the lure. Our lure was programmed to turn on at sunset and turn off at sunrise. The bat detectors were programmed to record from 30 min prior to sunset until 30 min after sunrise. The detectors recorded in full spectrum mode at a sampling rate of 256 kHz and settings were programmed according to the manufacturer’s recommendations. To confirm nightly operation of UV lures, we used two trail cameras (Bushnell model 119425C2, Overland Park, KS, USA) at each site with each camera programmed to take a picture of the lure every 15 min. We also used the trail camera pictures to help determine if it rained at a site. If it rained for more than a few hours or there was a technical malfunction, the experiment was repeated on the subsequent night until two nights of each before, during, and after conditions were achieved.

### 2.4. Analysis

We identified acoustic data in accordance with U.S. Fish and Wildlife Service approved guidelines [41,42] using Kaleidoscope Pro Version 5.4.0 with the Bats of North America 5.4.0 classifier automated identification software (Wildlife Acoustics, Inc., Maynard, MA, USA). For identification analyses, we considered the species listed in the study area description section as known or possibly occurring at PRWI and MCBQ, and all were included in the analysis. We used the 0 balanced/neutral setting with a minimum number of pulses set at 2. We only accepted nightly pass counts if the Maximum Likelihood Estimate (MLE) *p*-value was ≤0.05 for identification certainty [5]. Once all bat passes were identified to species or NOID (a bat present but not identified to species), we then reduced the sample to feeding buzzes from the larger pool of echolocation passes. To identify the bat feeding buzzes, we used a custom feeding buzz detection function developed in Program R [43]. We included both passes identified as species and classified as NOID because they could also contain feeding buzzes. We used Program R (version 4.2.2; R Foundation for Statistical Computing, Vienna, Austria) to remove any call sequences identified as noise by Kaleidoscope Pro and then ran the data through the buzz detector function using a buzz probability setting of 0.7. The buzz probability setting was a classification probability threshold to determine the cutoff for counting a sequence as a buzz call or not. A setting of 0 would result in any sequence being counted and a setting of 1 would result in no sequences being counted as a buzz. We found a setting of 0.7 to be a suitable setting based on testing and visual vetting of a small test dataset comprising a few passes taken from each site. The function was able to identify multiple feeding buzz sequences per recording file if there were more than one. The function outputs a .txt file containing the filename of a recording file that is identified as containing a buzz call, the time within the file that the buzz starts, and a classification certainty probability that the detected pulse sequence is in fact a buzz call. We retained only buzz passes that had a certainty probability of 0.7 or greater.

Because weather conditions and the day of the year are important determinants of bat activity, we collected weather data and combined it with our echolocation and feeding buzz data [44]. Hourly weather data from Aviation Routine Weather Reports recorded at Automated Surface Observing System (ASOS) stations was downloaded from the closest station, which was located at Marine Corps Airfield Quantico [45]. In a few instances, data was not available from the Quantico ASOS station, so we downloaded data from the next closest station, located at the Manassas Regional Airport [45]. We calculated nightly hourly averages for wind speed, temperature, and the proportion of a night with measurable precipitation (hereafter, precipitation). The fraction of the moon illuminated at midnight over the study area (hereafter called moon illumination) was downloaded from the Astronomical Applications Department of the U.S. Naval Observatory, Washington, DC [46]. We then converted the night date to the day of the year (yday) that coding the date from 1 to 365, and included a linear, quadratic, and cubic day of the year to account for non-linear changes in bat activity throughout the sampling period. A dummy variable was created for the UV lure treatment, with 1 corresponding to the control condition, 2 corresponding to the treatment with the lure activated, and 3 corresponding to the after-treatment condition with the lure off. Finally, because we had two detectors at the center (2021 only), 10 m, and 100 m positions for redundancy to protect from equipment malfunctions, we averaged and rounded the nightly bat activity data (total and species-specific nightly number of echolocation call sequences recorded at each site) for each of the three detector positions.

Prior to testing for UV lure effects, we determined if any covariates were correlated using the *corrplot* package (version 0.93) in R. Next, we examined our response variables to test for overdispersion and zero-inflation using the *performance* R package (version 0.10.8). We chose to include precipitation, wind speed, temperature, moon illumination, and yday for inclusion in the final models. We then used the *glmmTMB* package (version 1.1.8) in R to run 10 a priori selected generalized linear mixed models (GLMMs) with a negative binomial distribution (nbinom2) to model the effects of lure treatment, detector position, and environmental variables on overall echolocation activity (nightly total number of bat echolocation call sequences recorded at each site), species-dependent echolocation activity, and feeding activity (nightly total number of feeding buzz call sequences recorded) (Table 1). We set the reference position for the lure treatment variable as the initial two nights of the lure being off, before turning it on which acted as the control for lure treatment. The reference condition for detector position was set as the 100 m detector position. The 100 m detector position acted as the within-night control and control for the detector position variable. For the species that had heavily zero-skewed data, we used zero-inflated GLMMs with a negative binomial distribution (nbinom2). Site was included as a random effect, and polynomial terms were included for yday (yday, yday^2^, and yday^3^) to account for possible non-linear changes in bat activity throughout the summer. We then used Akaike Information Criterion corrected for small sample size (AICc; R package *AICcmodavg* version 2.3-3) to rank our models and chose the top model based on the AICc score, AICc weight, and the number of parameters [47]. For models that were within 2 delta AICc of the top model, we selected the most parsimonious model that included the lure treatment effect [17,47]. Models that did not converge were not included in the final model set. We used the R package *DHARMa* (version 0.4.6) to check model performance. Because we were interested in testing specific combinations of our treatment data, we performed preplanned orthogonal contrasts using the *emmeans* package (version 1.10.0) when the selected top model included the treatment effects and/or interaction term. If both lure and detector position were included in the top model, the preplanned contrasts included a test of lure effect (Lure OFF, BEFORE treatment “control conditions” at the CENTER detector versus Lure ON at the CENTER detector), a test of potential lag effects (Lure OFF, BEFORE treatment “control conditions” at the CENTER detector versus Lure OFF, AFTER treatment at the CENTER detector), a test to determine if the best position for receiving increased passes is slightly off to the side of the lure (10 m positions) or right at the lure device (center position) (Lure ON CENTER detector versus Lure ON 10 m detector), and lastly a test to determine if bat activity increased outside the illuminated area, which included the center and 10 m position detectors (Lure ON CENTER + 10 m detectors versus Lure ON 100 m detectors). When the lure treatment alone was included in the top model, our preplanned contrasts consisted of a test of lure effect (Lure OFF, BEFORE treatment versus Lure ON) and a test of potential lag effects (Lure OFF, BEFORE treatment versus Lure OFF, AFTER treatment). We performed an additional post hoc contrast for northern long-eared bats to compare the control conditions at the 100 m detectors to the lure on treatment at the 100 m detectors. This was performed to assess if there was an increase in northern long-eared bat passes outside the illuminated area at the 100 m detector positions compared to the lure on treatment condition Finally, we calculated the effect size of significant variables from the top model using estimated marginal means with 95% confidence intervals.

## 3. Results

### 3.1. Sampling Summary

We sampled a total of 118 nights across 22 sites. In 2020, 73 nights were sampled across 14 sites between 6 June and 16 August. In 2021, forty-five nights and eight sites were sampled between 19 June and 2 August. Some nights were excluded because the UV lure device and bat detector batteries failed, or the bat detector memory card was corrupted. Our final analysis dataset included 22 sites with 124 detector nights (one detector recording for one night) of the UV lure-off (before-treatment), 120 nights of our treatment condition with the UV lure activated, and 108 nights of the lure off (after treatment). Automated software identified 70,373 bat call sequences to species, with 64,555 sequences remaining after adjustment with the MLE *p*-value. The software also identified 84,688 call sequences as NOID, which were included along with echolocation passes in the feeding buzz analysis. The buzz detector function identified 144,962 feeding buzz call sequences after adjusting with the buzz probability threshold.

### 3.2. Total Echolocation Activity

The top model for total echolocation activity across all bat species combined was the lure treatment and weather model (Table 2). Although the weather-only model was a competing model, we selected the former model because we were primarily interested in the lure effect. Within the top model, the lure-off, after-treatment condition was significant along with the proportion of the night with measurable precipitation and the cubic day of the year variable. The lure-off, after-treatment condition had a slight positive effect on the total echolocation activity recorded. Precipitation also had a negative effect on total echolocation activity. The contrasts for lure effect and lag effect were both non-significant.

### 3.3. Species Specific Echolocation Activity

**Figure 6:**
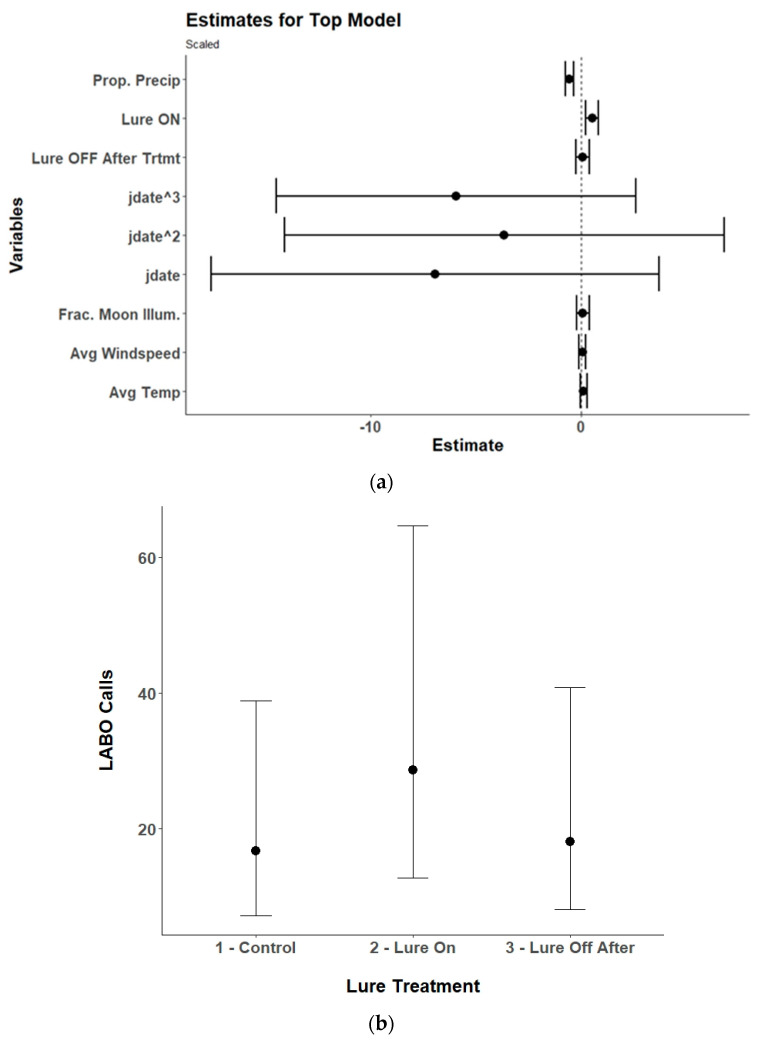
(**a**) Model estimates and 95% confidence intervals from the selected top generalized linear mixed model for eastern red bats (*Lasiurus borealis*; LABO) showing the response of received eastern red bat echolocation passes to the UV light lure treatments (control/lure off before treatment, lure on, and lure off after treatment), environmental variables, and the linear (yday), quadratic (yday^2^), and cubic (yday^3^) day of the year at Prince William Forest Park and Marine Corps Base Quantico, VA, USA from 2020 to 2021. Environmental variables included proportion of the night with measurable precipitation (Prop. Precip), average nightly temperature (Avg. Temp), average nightly windspeed (Avg. Windspeed), and the fraction of the moon illuminated at midnight (Frac. Moon Illum.). All numeric variables are centered and scaled. (**b**) Estimated marginal means and 95% confidence intervals from the top selected model for eastern red bats showing model predictions for eastern red bat echolocation passes during the three different UV lure treatments conditions. Conditions included light off before treatment (control), light on, and light off after treatment.

**Figure 7:**
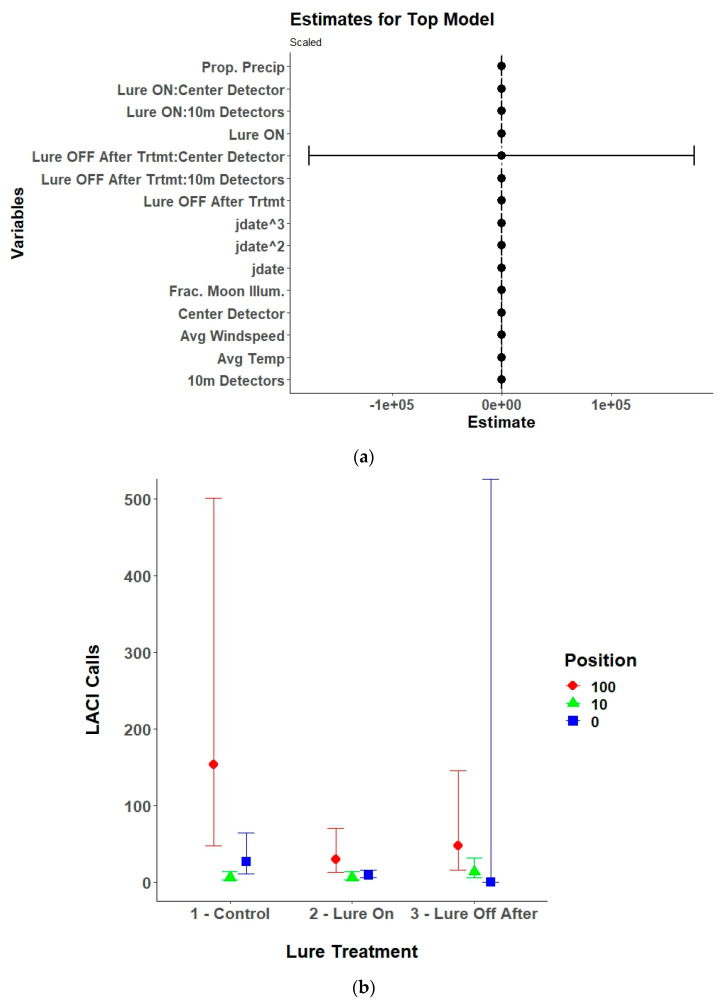
(**a**) Model estimates and 95% confidence intervals from the selected top generalized linear mixed model for North American hoary bats (*Lasiurus cinereus*; LACI) showing the response of received North American hoary bat echolocation passes to the interaction of UV light lure treatments (control/lure off before treatment, lure on, and lure off after treatment) with detector position (center/0 m, 10 m, and 100 m), along with environmental variables, and the linear (yday), quadratic (yday^2^), and cubic (yday^3^) day of the year at Prince William Forest Park and Marine Corps Base Quantico, VA, USA from 2020 to 2021. Environmental variables included proportion of the night with measurable precipitation (Prop. Precip), average nightly temperature (Avg. Temp), average nightly windspeed (Avg. Windspeed), and the fraction of the moon illuminated at midnight (Frac. Moon Illum.). All numeric variables are centered and scaled. (**b**) Estimated marginal means and 95% confidence intervals from the top selected model for North American hoary bats showing model predictions for North American hoary bat echolocation passes during the three different UV lure treatments conditions at the three different detector positions. Lure treatment conditions included light off before treatment (control), light on, and light off after treatment. Detector positions included the center/0 m, 10 m, and 100 m positions.

**Figure 8:**
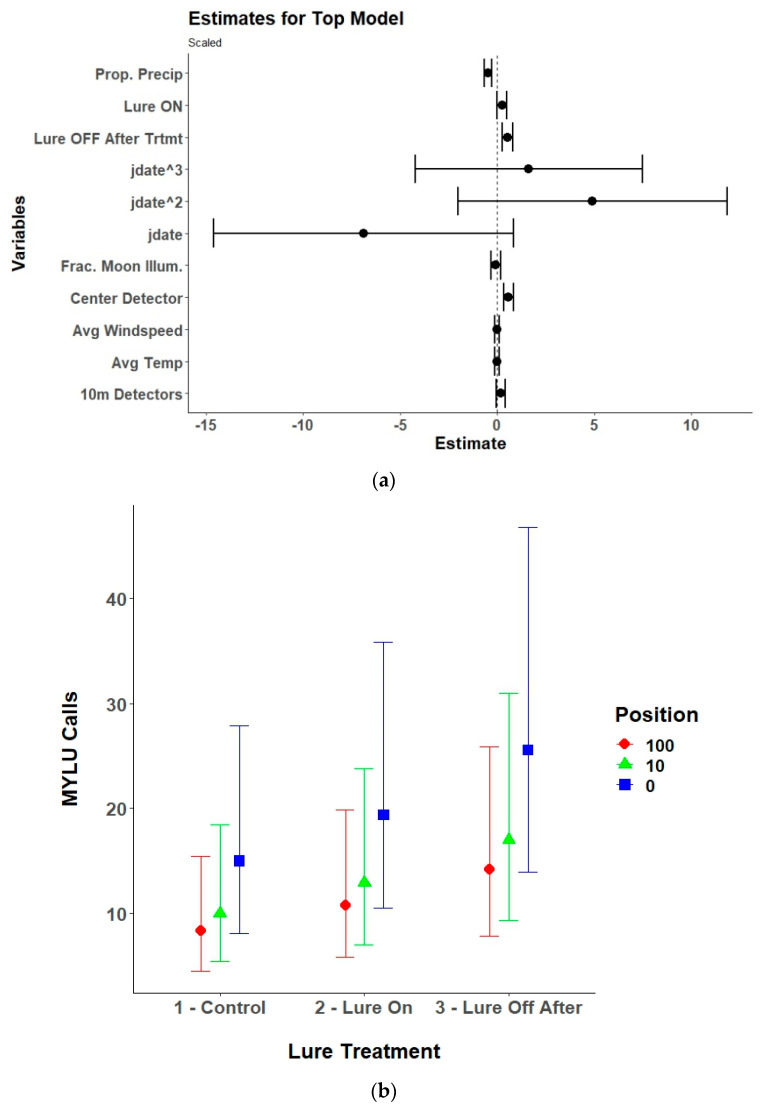
(**a**) Model estimates and 95% confidence inervals from the selected top generalized linear mixed model for little brown bats (*Myotis lucifugus*; MYLU) showing the response of received little brown bat echolocation passes to the UV light lure treatments (control/lure off before treatment, lure on, and lure off after treatment), detector position (center/0 m, 10 m, and 100 m), environmental variables, and the linear (yday), quadratic (yday^2^), and cubic (yday^3^) day of the year at Prince William Forest Park and Marine Corps Base Quantico, VA, USA from 2020 to 2021. Environmental variables included proportion of the night with measurable precipitation (Prop. Precip), average nightly temperature (Avg. Temp), average nightly windspeed (Avg. Windspeed), and the fraction of the moon illuminated at midnight (Frac. Moon Illum.). All numeric variables are centered and scaled. (**b**) Estimated marginal means and 95% confidence intervals from the top selected model for little brown bats showing model predictions for little brown bat echolocation passes during the three different UV lure treatments conditions at the three different detector positions. Lure treatment conditions included light off before treatment (control), light on, and light off after treatment. Detector positions included the center/0 m, 10 m, and 100 m positions.

**Figure 9:**
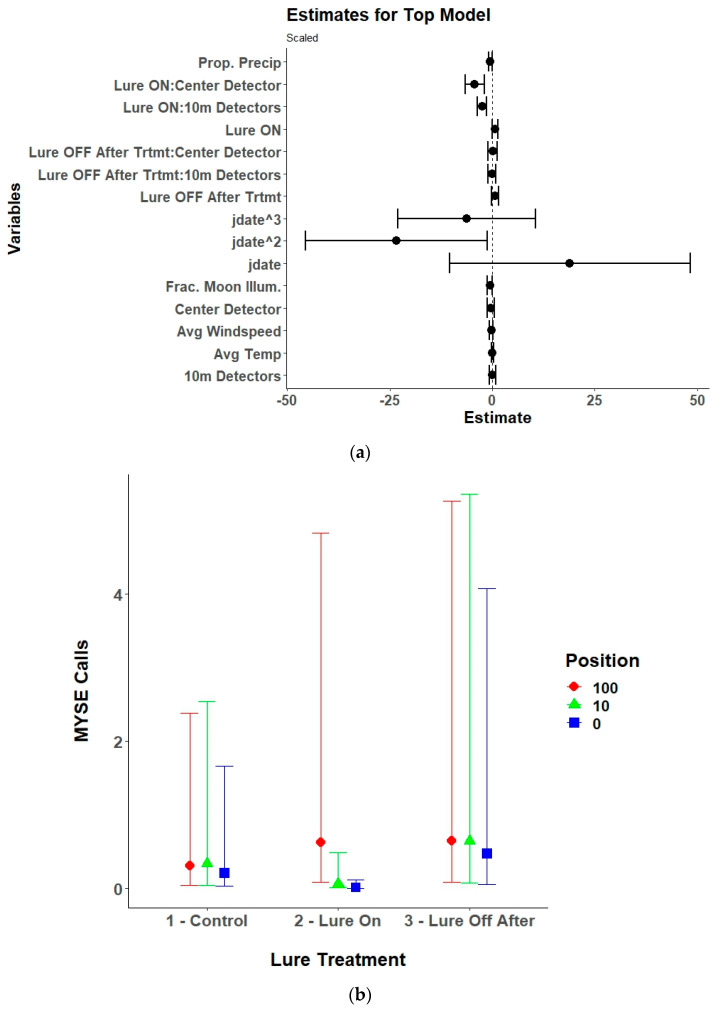
(**a**) Model estimates and 95% confidence intervals from the selected top generalized linear mixed model for northern long-eared bats (*Myotis septentrionalis*; MYSE) showing the response of received northern long-eared bat echolocation passes to the interaction of UV light lure treatments (control/lure off before treatment, lure on, and lure off after treatment) with detector position (center/0 m, 10 m, and 100 m), along with environmental variables, and the linear (yday), quadratic (yday^2^), and cubic (yday^3^) day of the year at Prince William Forest Park and Marine Corps Base Quantico, VA, USA from 2020 to 2021. Environmental variables included proportion of the night with measurable precipitation (Prop. Precip), average nightly temperature (Avg. Temp), average nightly windspeed (Avg. Windspeed), and the fraction of the moon illuminated at midnight (Frac. Moon Illum.). All numeric variables are centered and scaled. (**b**) Estimated marginal means and 95% confidence intervals from the top selected model for northern long-eared bats showing model predictions for northern long-eared bat echolocation passes during the three different UV lure treatments conditions at the three different detector positions. Lure treatment conditions included light off before treatment (control), light on, and light off after treatment. Detector positions included the center/0 m, 10 m, and 100 m positions.

**Figure 10:**
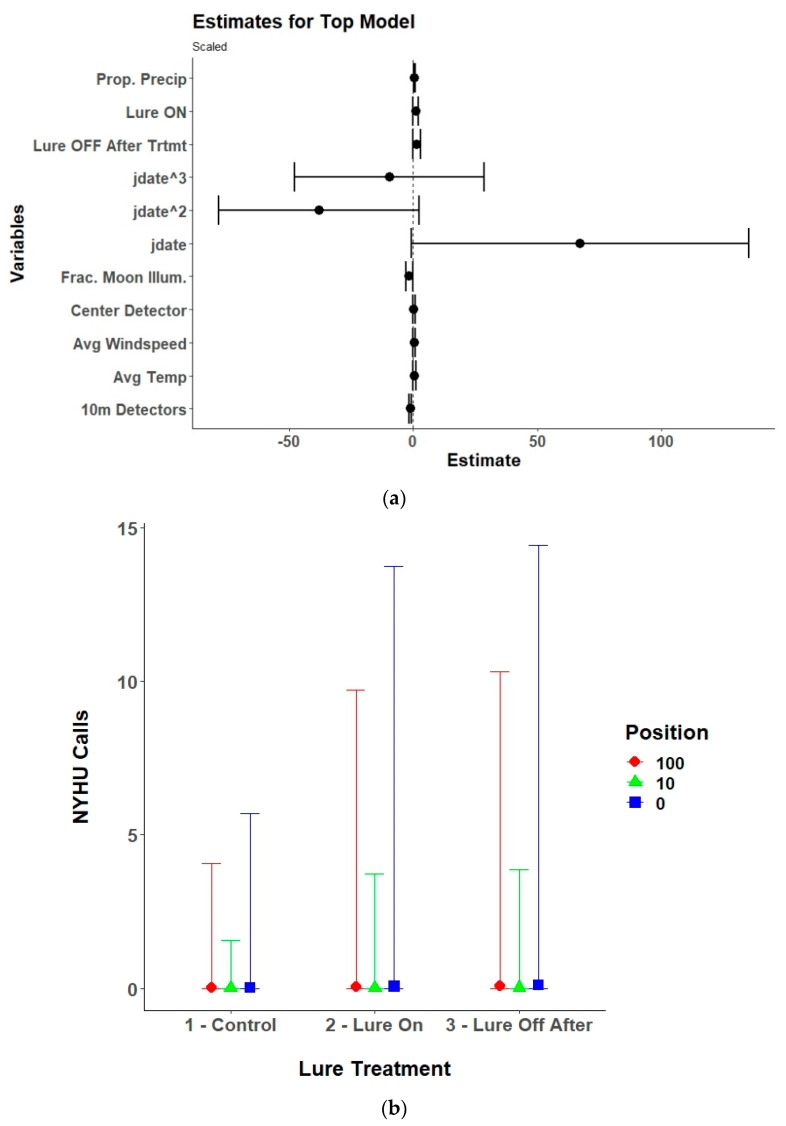
(**a**) Model estimates and 95% confidence intervals from the selected top generalized linear mixed model for evening bats (*Nycticeus humeralis*; NYHU) showing the response of received evening bat echolocation passes to the UV light lure treatments (control/lure off before treatment, lure on, and lure off after treatment), detector position (center/0 m, 10 m, and 100 m), environmental variables, and the linear (yday), quadratic (yday^2^), and cubic (yday^3^) day of the year at Prince William Forest Park and Marine Corps Base Quantico, VA, USA from 2020 to 2021. Environmental variables included proportion of the night with measurable precipitation (Prop. Precip), average nightly temperature (Avg. Temp), average nightly windspeed (Avg. Windspeed), and the fraction of the moon illuminated at midnight (Frac. Moon Illum.). All numeric variables are centered and scaled. (**b**) Estimated marginal means and 95% confidence intervals from the top selected model for evening bats showing model predictions for evening bat echolocation passes during the three different UV lure treatments conditions at the three different detector positions. Lure treatment conditions included light off before treatment (control), light on, and light off after treatment. Detector positions included the center/0 m, 10 m, and 100 m positions.

Lure treatment alone, lure treatment and detector position, or an interaction between the two variables was in the top model for six of the nine species of bats included in the analysis and lure treatment had a significant effect on five of the species (Table 2). The models for the silver-haired bat did not converge due to a low number of passes recorded and were excluded from the final results. The top model for Brazilian free-tailed bats was the null model and therefore also was excluded from the final results. For eastern red bats, the lure increased passes compared to control conditions with no lag effect (Table 3, Figure 6). The results for hoary bats should be interpreted cautiously due to the low number of passes received and generally poor performance of the top model. However, there was an overall decrease in hoary bat passes when the lure was activated, which continued after the lure was turned off. The interaction effects and contrasts indicate that there was higher activity at the 100 m detectors during all treatment conditions (Table A1, Figure 7). For little brown bats, the lure increased passes and the best position for recording those passes was at the center detector (Table A2, Figure 8). There was also a lag effect for little brown bats with higher call numbers than control conditions after the lure was turned off. Northern long-eared bats exhibited a more nuanced response to the lure with a decrease in passes received within the illuminated area (center and 10 m detectors) when the lure was activated, but a slight increase in passes at the 100 m detectors when the lure was activated (Table A3, Figure 9). The post hoc contrast indicated that there was a significant (*p* = 0.05) increase in northern long-eared bat echolocation passes at the 100 m detector positions from the control condition to the lure-on treatment condition. For evening bats, although the effects appear relatively small, the lure increased passes compared to control conditions, and there appeared to be a lag effect, with passes remaining slightly elevated as compared to control conditions (Table A4, Figure 10). The center detector appeared to record more passes than the 10 m detectors as there was a decrease in passes at the 10 m detector whereas the center detector showed no significant change from control conditions.

Moon illumination (fraction of the moon illuminated at midnight over the study area) and weather variables were included in the top model for eastern red bats, hoary bats, little brown bats, northern long-eared bats, evening bats, and tricolored bats. Moon illumination was significant for tricolored bats, showing a slight increase in passes with increasing moon illumination. Although it only approached significance, there was a decrease in northern long-eared bat passes with increasing moon illumination. Weather was an important predictor of bat echolocation activity with precipitation (proportion of the night with measurable precipitation) generally being the most influential. There was a negative relationship between precipitation and received echolocation passes for eastern red bats, little brown bats, and northern long-eared bats. Evening bats had a slightly positive relationship with precipitation, temperature, and windspeed. Windspeed was also significant for hoary bats with a negative relationship. Finally, there was a slight positive relationship between temperature and received passes for the tricolored bat.

### 3.4. Feeding Activity

**Figure 11:**
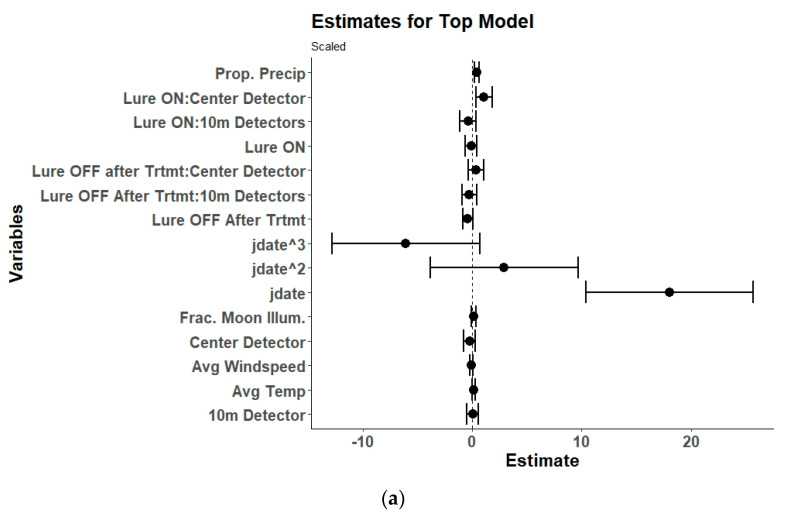

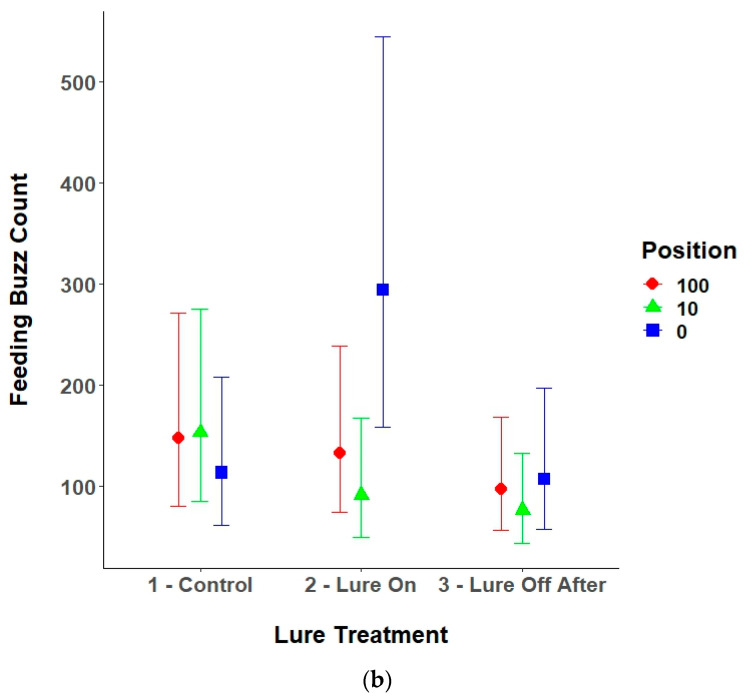
(**a**) Model estimates and 95% confidence intervals from the selected top generalized linear mixed model for bat feeding buzzes showing the response of received feeding buzz passes to the interaction of UV light lure treatments (control/lure off before treatment, lure on, and lure off after treatment) with detector position (center/0 m, 10 m, and 100 m), along with environmental variables, and the linear (yday), quadratic (yday^2^), and cubic (yday^3^) day of the year at Prince William Forest Park and Marine Corps Base Quantico, VA, USA from 2020 to 2021. Environmental variables included proportion of the night with measurable precipitation (Prop. Precip), average nightly temperature (Avg. Temp), average nightly windspeed (Avg. Windspeed), and the fraction of the moon illuminated at midnight (Frac. Moon Illum.). All numeric variables are centered and scaled. (**b**) Estimated marginal means and 95% confidence intervals from the top selected model for feeding buzzes showing model predictions for feeding buzz passes during the three different UV lure treatments conditions at the three different detector positions. Lure treatment conditions included light off before treatment (control), light on, and light off after treatment. Detector positions included the center/0 m, 10 m, and 100 m positions.

The top model for all bat feeding activity was the global model with an interaction between lure treatment and detector position (Table 4). There were no other competing models within two AICc of the top model. Within the top model, the interaction of the lure on treatment at the center detector position and the 10 m detector position were significant along with the proportion of the night with precipitation and day of the year (yday) (Table 4, Figure 11). The interaction of the activated UV lure at the center detector position significantly and substantially increased bat feeding activity. This increase was accompanied with a significant decrease in feeding activity received at the 10 m detectors while the lure was activated. There was no indication of a lag effect. There was a slight positive effect of precipitation on feeding activity. Finally, bat feeding activity increased throughout the sampling period regardless of lure or detector position.

## 4. Discussion

Use of the UV light lure increased echolocation activity of certain species in the trial including the eastern red bat, northern long-eared bat, evening bat, and the little brown bat but in nuanced ways for each species. Feeding activity, as measured by received feeding buzz passes, also increased when the lure was activated. Still, the overall recorded echolocation activity (all species combined) was not increased with use of the lure contrary to our predictions. Instead, use of the lure exhibited a species-specific effect with certain species increasing or decreasing during the lure treatment and afterwards. The eastern red bat had the strongest positive response to the lure. The eastern red bat is an edge-foraging species that feeds primarily on nocturnal lepidoptera, which are typically one of the most common insect species attracted to UV lights during entomological surveys [27]. Eastern red bats are also one of the two most common species captured during mist-net surveys that were conducted in the study area from 2017 to 2023 as a part of a separate study with big brown bats being the second most common species [39,40]. Despite being relatively uncommon in our study area and a relatively recent member of the bat assemblage [7,48], evening bat echolocation activity also increased around the lure. This response also could be attributable in part to their edge-foraging strategies [49]. The roads and stream corridors where lure sites were placed are included in their typical foraging habitat [50] and we were able to concentrate the bats there using the lure. Evening bats are also known to forage along the horizontal edge just above the canopy line [49]. The insect aggregation created by the lure could have drawn evening bats down lower to the point where they were able to be detected by the acoustic recorders. Although little brown bat echolocation activity appeared to increase with use of the UV lure, there have been no mist-net captures of this species in the study area in recent years and the nearest known contemporary captures occur approximately 200 km upstream on the Potomac River [7,51]. It is possible that these are misclassifications of eastern red bats as has been suggested previously [4,52]. However, the U.S. Geological Survey and U.S. Fish and Wildlife software test results for the version of Kaleidoscope Pro that we used did not indicate any issues with misclassification of little brown bats as eastern red bats [41]. Without careful hand-vetting of acoustic passes, it is difficult to draw any conclusions as to why this species was recorded but never captured. Little brown bats are also primarily an edge-foraging species, albeit more riparian zone biased [53,54]. The results indicate that our prediction that the lure would have the greatest effect attracting edge-foraging species is likely correct. 

Open-space foraging species were either not attracted to the lure (big brown bat) or showed a negative response to the lure (hoary bat). No effect of the lure was surprising for big brown bats since they are the second most captured bat during mist-net surveys in the study area [39,40]. However, it is possible that big brown bats were captured during mist-net surveys while commuting from roosting to foraging habitat rather than actively foraging along the roads and trails where UV lure sites were located. Although hoary bats appeared to show a negative response to the UV lure, the fit of the top model was poor, and few hoary bat passes were recorded. The low number of passes was likely due to hoary bats primarily foraging above the forest canopy, out of range of the detectors, and they are relatively uncommon in the study area during the summer [8,49,55]. It may be possible that the UV lights were drawing lepidopterans down and away from the upper levels of the forest canopy, thereby actually reducing the food resources available for hoary bats in the immediate vicinity of the lure site. Hoary bats are adapted for open-space foraging with a high wing loading and high wing to body size aspect ratio allowing for fast flight at high altitudes rather than slow, maneuverable flight in cluttered conditions. Hoary bats also use long, constant-frequency echolocation calls at a low frequency for increased range in the open but at the expense of resolution in high clutter environments [49,56,57]. These morphological and physiological adaptations of hoary bats may have precluded them from being able to effectively take advantage of insect aggregation at the lower levels of the forest where the UV lure and bat detectors were deployed. Accordingly, we suggest testing UV light lures in different foraging habitat types including open spaces, edge habitat, and forest interiors to capture inherent differences in the feeding strategies of different species of bats. 

The results for the northern long-eared bat are particularly interesting because echolocation activity overall increased slightly when the lure was activated but strongly decreased at the detectors within the illuminated area located near the lure device (center and 10 m units). Activity returned to being equitable between the detector positions after the lure was turned off, albeit at a slightly higher level than control conditions. This may be due to northern long-eared bats being a forest interior foraging and more light-averse species [6,19,58]. We observed that the UV lure lights did emit a substantial amount of visible light in the purple and blue range. Although we used the uncoated UV lights in an attempt to maximize the attraction of different insect species, the remaining visible light may have deterred northern long-eared bats from entering the illuminated area and taking advantage of the insect swarm around the lights. Alternatively, other species foraging around the lure, such as the eastern red bat, may have competitively excluded northern long-eared bats from feeding on the insect swarm [34,59,60,61]. It remains unknown whether the slight increase in received northern long-eared bat passes was due to attraction to the area, but exclusion from the center of the site. Alternatively, the increase could be simply due to a small increase in detections as northern long-eared bats were pushed from the center of the site. Based on the finding that northern long-eared bats seemed to avoid the illuminated area due to potential light aversion or competitive exclusion, we suggest caution when using UV lights for surveys. We also advise caution when attempting to provide a foraging patch for WNS-impacted bat species as was tested by Frick et al. [17], because not all species may benefit. 

The UV light lure significantly increased overall feeding activity around the lure device as noted by others [16,17,30,31,32,33,34]. Feeding activity was measured by received feeding buzzes from all species combined. This indicates that the light was successful at attracting insect species that bats prey on and that bats were able to find and take advantage of the artificial insect swarm. We observed large swarms of insects including Lepidoptera congregating around the lights. Interestingly, moths were observed sitting on the UV lure device lights, rain covers, and rope during the day as well. Examination of the model output, estimates, and contrasts indicates that the best position for detecting feeding buzzes in conjunction with a UV light lure device is right at the device instead of off to either side by 10 m. Both the center and 10 m detectors experienced an increase in passes; however, the increase was substantially greater at the center detector. The contrast between the 10 m detectors and the center detectors during lure treatment was significant which further indicates a difference between the two positions for receiving feeding buzz passes. It is noteworthy that the lure effect was not significant for overall echolocation passes combined but was significant for overall feeding buzzes. It is possible that feeding buzzes were the predominant call type produced by bats flying around the lure instead of search-phase echolocation passes that are used for species identification. This inability to positively identify a bat echolocation pass to species represents an inherent limitation of using UV lights during acoustic surveys at present. We attempted to ameliorate this problem by using a linear array of bat detectors along the most obvious flyway in the hopes we would record bats as they fly towards the lure. However, other than the northern long-eared bat that was possibly repelled by the light, echolocation passes primarily increased at the center detector placed right next to the UV light. Future work could include an analysis of call quality to investigate if use of a UV lure potentially degrades echolocation call sequences by causing bats to use more feeding-type calls that are more difficult to identify to species. Overall, the feeding activity results indicate that UV light lure devices do attract bats to feed around the light.

For both echolocation activity and feeding activity, weather had a strong influence independent of the lure effect. The precipitation variable which was the proportion of the night with measurable precipitation was generally a significant predictor of echolocation and feeding activity. Although in most cases we were able to sample on nights with none to minimal precipitation, there were nights that did experience substantial precipitation. Precipitation had a strong negative effect for eastern red bats, little brown bats, northern long-eared bats, and overall feeding activity. Temperature has been shown to be an important variable influencing bat activity on the landscape [17,44]. However, this variable had minimal influence on our study in that the sampling period was during the summer, when the average daily temperatures stayed fairly consistent and warm. Windspeed has also been shown to decrease bat activity, but only the hoary bat model indicated a significant negative relationship between echolocation activity and increasing windspeed. Although the lure sites were corridors through forest with an internal edge, they were likely not open enough for hoary bats but were sheltered enough to prevent wind from strongly affecting the edge-foraging and clutter-foraging bats recorded at the sites. 

We predicted that moon illumination would also influence the effectiveness of the UV light lure as entomology research indicates that brightly lit conditions decrease the attraction effectiveness of UV light traps [62]. Moon illumination was either not included in the top model or was not significant for overall and species-specific echolocation activity nor for feeding activity except for the tricolored bat. The tricolored bat showed a slight increase in echolocation activity with increasing moon illumination. The overall unimportance of moon illumination may be due to our site selection. Despite being along road and stream corridors, the sites were still largely under mature forest canopies whereby moon illumination effects would have been moderated or negated.

We chose to use full-spectrum bat detectors because this recording type is considered to be more “sensitive” and better able to record multiple bats calling on top of each other [63,64,65,66]. We suspected that if the lure device worked, there could be many bats all calling at the same time. Although we did not analyze the number of call sequences that contained more than one bat, we did observe numerous call sequences with more than one bat in them. The choice of full-spectrum bat detectors came at a cost in that lure use increased received passes and insect noise, causing our bat detector batteries and memory cards to reach storage capacity faster than expected, particularly in the first year of the study. Moreover, full-spectrum detectors generally are more power and memory space-consuming than a zero-crossing/frequency division-type bat detector [66]. That forced us to eliminate some nights from analysis. The feeding buzz detection R script worked well overall, but broadband noise in the recording files did impact results. For example, the function would sometimes identify repetitive broadband insect or environmental noise as a feeding buzz (false positive) and sometimes miss a faint feeding buzz (false negative). We were able to eliminate most false positives and false negatives in the feeding buzz detection results by using conservative settings and thresholds, but invariably, there were still some errors observed in the results. We suggest that future work carefully consider detector settings and pre-filtering of data before running it through the buzz detector function to help lessen broadband noise within the recording files. Further work developing tools to identify feeding buzzes could be important for future work involving UV lure development, as well as for other reasons such as identifying important foraging habitats [44].

Although our study did improve upon the limited sample sizes of past studies, future studies sampling a wider range of geographic locations, habitat types, and clutter condition types (e.g., high-clutter forest interior vs. low-clutter open field) would help add clarity. The primary interest in using a lure device during surveys would be to increase detection of rare, threatened, and endangered species. Despite northern long-eared bats comprising a minority of received echolocation passes during the study, as well as a minority of physical captures during mist-net surveys conducted in the study area, the study demonstrated an increase in echolocation activity of the species with use of a UV lure [39,40]. The study showed that UV lures can increase the number of received echolocation call sequences, but future studies could incorporate an assessment of received call quality with and without a UV lure device. Improved call quality in addition to more received passes would help automatic identification programs to identify bat passes to species with a higher degree of certainty and could therefore improve species richness determinations during bat biodiversity inventories similar to the differences in measured richness between mist-net and standard acoustic surveys [9,67,68]. Species accumulation curve analyses could also be helpful to determine if acoustic surveys using UV lures can achieve an acceptable level of certainty with less time and/or fewer detectors on the landscape than traditional acoustic surveys [69,70]. 

The sampling nights after the lure was turned off, as well as placing detectors outside the illuminated area, proved to provide important information on any lag attraction and/or light-averse behaviors. Based on the northern long-eared bat results from those sampling periods and detector positions, future testing might consider including on and off periods throughout a single sampling night or alternating on and off nights to perhaps allow light-averse species to take advantage of insect aggregations. It may also be important to deploy an array or grid of bat detectors, as we did, to capture activity beyond the illuminated area. However, within the illuminated area, our results show that deploying a bat detector as close as possible to the UV lights is important. Finally, our UV light lure device worked well and achieved our goal of building a programmable, field-deployable lure device. It operated reliably as indicated by examining our camera trap pictures, remained reasonably waterproof (although could not withstand a flood event), and was relatively easy to program and deploy. However, our design was heavy (requiring eight 12-volt batteries), and the glass UV fluorescent tubes were prone to breakage. Future UV light lures that use LEDs would require less power, would be more robust, could be housed in a waterproof enclosure, and likely could be made more cheaply. Inexpensive LED UV lights are readily available; however, those lights tend to emit a narrower range of UV light as compared to the UV fluorescent tubes along with a substantial amount of visible light [27]. Multiple LEDs likely need to be used that have different peak wavelengths of emitted UV light. Filters, such as the dark purple coating used on black light blue (BLB) UV fluorescent tubes may also be a useful addition to further reduce visible light emission and lessen possible deterrent effects for light-averse bats. 

## 5. Conclusions

Our study was one of the first comprehensive attempts to use a UV light lure to increase the acoustic detection of bats. We demonstrated that UV lures show promise by increasing echolocation and feeding activity, including that of a federally endangered species. However, the response is species-dependent, with generally edge-foraging species showing the greatest response, and one species possibly being deterred from the illuminated area. More research is warranted with the hope of eventually using UV lures to reduce the time and effort required to survey for rare bat species. The results also corroborate similar efforts to use UV lights to improve foraging opportunities for bats impacted by White-Nose Syndrome as they enter hibernation in the fall and emerge in the spring [17].

## Figures and Tables

**Table 1:** List of the ten a priori generalized linear mixed models (GLMMs) used to model the effect of the UV light lure (Lure) and detector position (Distance) within a site along with environmental variables on received echolocation passes of bats at Prince William Forest Park and Marine Corps Base Quantico, VA, USA, 2020–2021. The environmental variables included fraction of the moon illuminated at midnight (Moon), proportion of the night with measurable precipitation (Precipitation), average nightly temperature (Temperature), average nightly windspeed (Windspeed), and day of the year (yday). Day of the year includes a linear, quadratic, and cubic term. Lure treatment consists of three conditions: lure off before treatment, lure on, lure off after treatment. Detection position (Distance) includes 100 m, 10 m, and 0 m (at the lure). A plus sign (+) indicates an additive effect and an asterisk (*) indicates an interactive effect.

Model Name	Formula
Null	~1
Global with Interaction	~Lure * Distance + Precipitation + Temperature + Windspeed + Moon + poly(yday, factor = 3) + (1|Site)
Global	~Lure + Distance + Precipitation + Temperature + Windspeed + Moon + poly(yday, factor = 3) + (1|Site)
Lure	~Lure + poly(yday, factor = 3) + (1|Site)
Distance	~Distance + poly(yday, factor = 3) + (1|Site)
Lure and Distance	~Lure + Distance + poly(yday, factor = 3) + (1|Site)
Lure and Distance Interaction	~Lure * Distance + poly(yday, factor = 3) + (1|Site)
Weather	~Precipitation + Temperature + Windspeed + Moon + poly(yday, factor = 3) + (1|Site)
Lure and Weather	~Lure + Precipitation + Temperature + Windspeed + Moon + poly(yday, factor = 3) + (1|Site)
Distance and Weather	~Distance + Precipitation + Temperature + Windspeed + Moon + poly(yday, factor = 3) + (1|Site)

**Table 2:** Top model selected for each species of bat (top), all species combined (middle), and feeding buzzes (bottom) using Akaike Information Criteria, corrected for small sample size (AICc) at Prince William Forest Park and Marine Corps Base Quantico, VA, USA, 2020–2021. We considered models that were within two delta AICc of the top model and then selected the most parsimonious model that included the lure treatment effect. Models that did not converge were not included in the final model set. For full model selection tables, see Table 3 and Appendix A. Models that included a significant effect of lure treatment (*p* < 0.05) are indicated by an asterisk.

Species	Top Model	ω_i_
Eastern red bat (*Lasiurus borealis*)	Lure and Weather *	0.82
Big brown bat (*Eptesicus fuscus*)	Lure	0.45
Little brown bat (*Myotis lucifugus*)	Global no interaction *	0.94
Northern long-eared bat (*Myotis septentrionalis*)	Global with interaction *	0.91
North American hoary bat (*Lasiurus cinereus*)	Global with interaction *	0.45
Indiana bat (*Myotis sodalis*)	Distance	0.51
Evening bat (*Nycticeus humeralis*)	Global no interaction *	0.37
Tricolored bat (*Perimyotis subflavus*)	Distance and weather	0.72
Silver-haired bat (*Lasionycteris noctivagans*)	did not converge	NA
Brazilian free-tailed bat (*Tadarida brasiliensis*)	Null	NA
All Species Combined	Lure and Weather	0.41
Feeding Buzzes	Global with Interaction *	0.94

* Indicates the UV lure had a significant effect in the model.

**Table 3:** Model AIC ranking table (top), model output for the selected top generalized linear mixed model (middle), and preplanned orthogonal contrasts (bottom) for the eastern red bat (*Lasiurus borealis*) showing the response of received eastern red bat echolocation passes to the UV lure treatments, environmental variables, and the linear (yday), quadratic (yday^2^), and cubic (yday^3^) day of the year at Prince William Forest Park and Marine Corps Base Quantico, VA, USA, 2020–2021. Environmental variables included proportion of the night with measurable precipitation (Prop. Precip), average nightly temperature (Avg. Temp), average nightly windspeed (Avg. Windspeed), and the fraction of the moon illuminated at midnight (Frac. Moon Illum.). All numeric variables are centered and scaled. The coefficient values (β), standard errors (SE), 95% lower (LCL) and upper (UCL) confidence levels, and *p* values are shown. The contrasts test preplanned comparisons between lure treatment levels.

Model			AICc		ΔAIC		ω_i_
Lure and Weather			2633.33		0.00		0.82
Global no Interaction			2637.07		3.74		0.13
Global with Interaction			2639.08		5.76		0.05
Weather Only			2643.93		10.61		<0.01
Distance and Weather			2647.66		14.34		<0.01
Lure Only			2652.95		19.62		<0.01
Lure and Distance			2656.08		22.76		<0.01
Lure, Distance, and Interaction			2657.30		23.97		<0.01
Distance Only			2676.86		43.53		<0.01
Null Model			2862.54		229.21		<0.01
Variables	β	SE		LCL		UCL	*p* Value
Lure ON	0.54	0.15		0.24		0.84	0.001
Lure OFF After Trtmt	0.08	0.17		−0.26		0.41	0.645
Prop. Precip	−0.55	0.10		−0.74		−0.36	<0.001
Avg. Temp	0.13	0.08		−0.03		0.29	0.106
Avg. Windspeed	0.07	0.08		−0.08		0.22	0.389
Frac. Moon Illum.	0.09	0.15		−0.21		0.40	0.547
yday	−6.93	5.42		−17.56		3.69	0.201
yday^2^	−3.65	5.33		−14.10		6.80	0.494
yday^3^	−5.93	4.36		−14.48		2.61	0.174
Preplanned Contrast	β	SE		LCL		UCL	*p*-value
Before Treatment, Lure OFF versus Lure ON	0.54	0.15		0.19		0.89	0.001
Before Treatment, Lure OFF versus After Treatment, Lure OFF	0.08	0.17		−0.30		0.46	0.645

**Table 4:** Model AIC ranking table (top), model output for the selected top generalized linear mixed model (middle), and preplanned orthogonal contrasts (bottom) for feeding buzzes showing the response of received feeding buzz passes to the UV lure treatments, detector position, an interaction between lure treatment and detector position, environmental variables, and the linear (yday), quadratic (yday^2^), and cubic (yday^3^) day of the year at Prince William Forest Park and Marine Corps Base Quantico, VA, USA 2020–2021. Environmental variables included proportion of the night with measurable precipitation (Prop. Precip), average nightly temperature (Avg. Temp), average nightly windspeed (Avg. Windspeed), and the fraction of the moon illuminated at midnight (Frac. Moon Illum.). All numeric variables are centered and scaled. The coefficient values (β), standard errors (SE), 95% lower (LCL) and upper (UCL) confidence levels, and *p* values are shown. The contrasts test preplanned comparisons between lure treatment levels and bat detector positions within a site.

Model			AICc		ΔAIC		ω_i_
Global with Interaction			5259.90		0.00		0.94
Global no Interaction			5266.78		6.88		0.03
Lure, Distance, and Interaction			5267.66		7.76		0.02
Lure and Weather			5269.53		9.63		0.01
Lure and Distance			5271.08		11.18		<0.01
Distance and Weather			5272.79		12.88		<0.01
Lure Only			5273.86		13.95		<0.01
Distance Only			5276.14		16.23		<0.01
Weather Only			5276.90		16.99		<0.01
Null Model			5522.56		262.66		<0.01
Variables	β	SE		LCL		UCL	*p* Value
Lure ON	−0.11	0.27		−0.63		0.42	0.694
Lure OFF After Trtmt	−0.41	0.25		−0.91		0.08	0.099
10 m Detector	0.04	0.27		−0.50		0.57	0.899
Center Detector	−0.26	0.27		−0.79		0.26	0.326
Prop. Precip	0.40	0.11		0.19		0.61	<0.001
Avg. Temp	0.12	0.08		−0.03		0.27	0.129
Avg. Windspeed	−0.12	0.07		−0.26		0.02	0.082
Frac. Moon Illum.	0.12	0.11		−0.09		0.33	0.265
yday	18.05	3.90		10.41		25.70	<0.001
yday^2^	2.90	3.46		−3.88		9.69	0.401
yday^3^	−6.09	3.44		−12.83		0.65	0.077
Lure ON:10 m Detectors	−0.41	0.38		−1.15		0.33	0.279
Lure OFF After Trtmt:10 m Detectors	−0.28	0.34		−0.94		0.39	0.413
Lure ON:Center Detector	1.06	0.38		0.31		1.81	0.006
Lure OFF after Trtmt:Center Detector	0.36	0.37		−0.36		1.07	0.331
Contrast	β	SE		LCL		UCL	*p* Value
Before Treatment, Lure OFF at Center Detector versus Lure ON at Center Detector	0.95	0.28		0.25		1.65	0.001
Before Treatment, Lure OFF at Center Detector versus After Treatment, Lure OFF at Center Detector	−0.06	0.28		−0.76		0.65	0.836
Lure ON at Center and 10 m Detectors versus Lure ON at 100 m Detector	−0.21	0.24		−0.81		0.39	0.511
Lure ON at Center Detector versus Lure ON at 10 m Detector	1.17	0.27		0.49		1.84	<0.001

## Data Availability

At the time of publication, data was not publicly available from the National Park Service nor the U.S. Marine Corps. Author Samuel R. Freeze (srfreeze@vt.edu) can be contacted for data availability.

## Appendix A

This appendix contains the species-specific model AIC ranking, model output, and orthogonal contrast tables from the Generalized Linear Mixed Models (GLMMs) used in this study and reported in Section 3.3. *Species Specific Echolocation Activity*.

**Table A1:** Model AIC ranking table (top), model output for the selected top generalized linear mixed model (middle), and preplanned orthogonal contrasts (bottom) for the hoary bat (*Lasiurus cinereus*) showing the response of received Hoary bat echolocation passes to the UV lure treatments, detector position, an interaction between lure treatment and detector position, environmental variables, and the linear (yday), quadratic (yday^2^), and cubic (yday^3^) day of the year at Prince William Forest Park and Marine Corps Base Quantico, VA, USA 2020–2021. Environmental variables included proportion of the night with measurable precipitation (Prop. Precip), average nightly temperature (Avg. Temp), average nightly windspeed (Avg. Windspeed), and the fraction of the moon illuminated at midnight (Frac. Moon Illum.). All numeric variables are centered and scaled. The coefficient values (β), standard errors (SE), 95% lower (LCL) and upper (UCL) confidence levels, and *p* values are shown. The contrasts test preplanned comparisons between lure treatment levels and bat detector positions within a site.

Model	AICc		ΔAIC		ω_i_
Global with Interaction	591.03		0.00		0.45
Lure, Distance, and Interaction	591.10		0.07		0.43
Distance Only	595.10		4.07		0.06
Lure and Distance	596.36		5.34		0.03
Global no Interaction	597.02		5.99		0.02
Distance and Weather	599.51		8.48		0.01
Lure Only	608.40		17.37		<0.01
Weather Only	609.09		18.06		<0.01
Lure and Weather	610.96		19.93		<0.01
Null Model	613.30		22.27		<0.01
Variables	β	SE	LCL	UCL	*p* Value
Lure ON	−1.64	0.58	−2.77	−0.51	0.005
Lure OFF After Trtmt	−1.18	0.60	−2.35	−0.01	0.048
10 m Detectors	−3.28	0.66	−4.58	−1.99	<0.001
Center Detector	−1.75	0.58	−2.89	−0.61	0.003
Prop. Precip	−0.01	0.26	−0.52	0.49	0.955
Avg. Temp	−0.03	0.18	−0.38	0.33	0.890
Avg. Windspeed	−0.50	0.19	−0.88	−0.12	0.009
Frac. Moon Illum.	0.29	0.24	−0.17	0.76	0.217
yday	−26.67	6.76	−39.93	−13.41	<0.001
yday^2^	−32.99	7.83	−48.33	−17.65	<0.001
yday^3^	−25.94	5.83	−37.37	−14.50	<0.001
Lure ON:10 m Detectors	1.64	0.87	−0.06	3.33	0.059
Lure OFF After Trtmt:10 m Detectors	2.00	0.85	0.33	3.67	0.019
Lure ON:Center Detector	0.58	0.68	−0.76	1.92	0.395
Lure OFF After Trtmt:Center Detector	−26.13	89,860.91	−176,150.29	176,098.02	1.000
Contrast	β	SE	LCL	UCL	*p* Value
Before Treatment, Lure OFF at Center Detector versus. Lure ON at Center Detector	−1.06	0.50	−2.30	0.19	0.068
Before Treatment, Lure OFF at Center Detector versus After Treatment, Lure OFF at Center Detector	−27.32	89,860.91	−224,473.41	224,418.78	1.000
Lure ON at Center and 10 m Detectors versus Lure ON at 100 m Detector	1.41	0.44	0.30	2.51	0.006
Lure ON at Center Detector versus Lure ON at 10 m Detector	0.47	0.47	−0.70	1.64	0.416

**Table A2:** Model AIC ranking table (top), model output for the selected top generalized linear mixed model (middle), and preplanned orthogonal contrasts (bottom) for the little brown bat (*Myotis lucifugus*) showing the response of received little brown bat echolocation passes to the UV lure treatments, detector position within a site, environmental variables, and the linear (yday), quadratic (yday^2^), and cubic (yday^3^) day of the year at Prince William Forest Park and Marine Corps Base Quantico, VA, USA, 2020–2021. Environmental variables included proportion of the night with measurable precipitation (Prop. Precip), average nightly temperature (Avg. Temp), average nightly windspeed (Avg. Windspeed), and the fraction of the moon illuminated at midnight (Frac. Moon Illum.). All numeric variables are centered and scaled. The coefficient values (β), standard errors (SE), 95% lower (LCL) and upper (UCL) confidence levels, and *p* values are shown. The contrasts test preplanned comparisons between lure treatment levels and bat detector positions within a site.

Model	AICc		ΔAIC		ω_i_
Global no Interaction	2500.38		0.00		0.94
Global with Interaction	2506.44		6.06		0.05
Distance and Weather	2510.23		9.85		0.01
Lure and Distance	2511.94		11.56		<0.01
Lure, Distance, and Interaction	2516.61		16.23		<0.01
Lure and Weather	2517.88		17.50		<0.01
Distance Only	2517.89		17.51		<0.01
Weather Only	2525.47		25.09		<0.01
Lure Only	2530.47		30.09		<0.01
Null Model	2703.18		202.80		<0.01
Variables	β	SE	LCL	UCL	*p* Value
Lure ON	0.26	0.13	0.01	0.51	0.044
Lure OFF After Trtmt	0.53	0.14	0.26	0.81	<0.001
10 m Detectors	0.18	0.12	−0.06	0.41	0.136
Center Detector	0.59	0.12	0.34	0.83	<0.001
Prop. Precip	−0.47	0.10	−0.67	−0.27	<0.001
Avg. Temp	0.00	0.06	−0.11	0.11	0.998
Avg. Windspeed	0.01	0.06	−0.11	0.12	0.889
Frac. Moon Illum.	−0.07	0.13	−0.32	0.18	0.576
yday	−6.87	3.94	−14.60	0.85	0.081
yday^2^	4.91	3.54	−2.02	11.84	0.165
yday^3^	1.62	2.98	−4.23	7.46	0.588
Preplanned Contrast	β	SE	LCL	UCL	*p* Value
Before Treatment, Lure OFF at Center Detector versus Lure ON at Center Detector	0.26	0.13	−0.06	0.58	0.044
Before Treatment, Lure OFF at Center Detector versus After Treatment, Lure OFF at Center Detector	0.53	0.14	0.18	0.88	0.001
Lure ON at Center and 10 m Detectors versus Lure ON at 100 m Detector	−0.38	0.10	−0.64	−0.12	0.001
Lure ON at Center Detector versus Lure ON at 10 m Detector	0.41	0.13	0.09	0.72	0.002

**Table A3:** Model AIC ranking table (top), model output for the selected top generalized linear mixed model (middle), and preplanned orthogonal contrasts with additional post hoc contrasts (bottom) for the northern long-eared bat (*Myotis septentrionalis*) showing the response of received northern long-eared bat echolocation passes to the UV lure treatments, detector position, an interaction between lure treatment and detector position, environmental variables, and the linear (yday), quadratic (yday^2^), and cubic (yday^3^) day of the year at Prince William Forest Park and Marine Corps Base Quantico, VA, USA, 2020–2021. Environmental variables included proportion of the night with measurable precipitation (Prop. Precip), average nightly temperature (Avg. Temp), average nightly windspeed (Avg. Windspeed), and the fraction of the moon illuminated at midnight (Frac. Moon Illum.). All numeric variables are centered and scaled. The coefficient values (β), standard errors (SE), 95% lower (LCL) and upper (UCL) confidence levels, and *p* values are shown. The contrasts test preplanned comparisons between lure treatment levels and bat detector positions within a site. An additional post hoc contrast shows a comparison between the “before treatment, lure off” condition at the 100 m detectors versus the “lure on” condition at the 100 m detectors.

Model	AICc		ΔAIC		ω_i_
Global with Interaction	690.16		0.00		0.91
Lure, Distance, and Interaction	694.68		4.52		0.09
Lure and Distance	712.45		22.29		<0.01
Global no Interaction	712.69		22.52		<0.01
Distance and Weather	714.78		24.62		<0.01
Weather Only	718.16		28.00		<0.01
Distance Only	718.46		28.30		<0.01
Lure and Weather	718.65		28.49		<0.01
Lure Only	721.09		30.93		<0.01
Null Model	822.17		132.01		<0.01
Variables	β	SE	LCL	UCL	*p* Value
Lure ON	0.71	0.35	0.03	1.38	0.041
Lure OFF After Trtmt	0.73	0.42	−0.10	1.56	0.084
10 m Detectors	0.08	0.40	−0.70	0.86	0.839
Center Detector	−0.41	0.45	−1.29	0.48	0.365
Prop. Precip	−0.43	0.18	−0.78	−0.08	0.016
Avg. Temp	0.08	0.14	−0.19	0.34	0.572
Avg. Windspeed	−0.23	0.19	−0.60	0.14	0.226
Frac. Moon Illum.	−0.56	0.31	−1.17	0.05	0.070
yday	18.96	14.99	−10.42	48.34	0.206
yday^2^	−23.33	11.32	−45.52	−1.14	0.039
yday^3^	−6.21	8.59	−23.05	10.62	0.470
Lure ON:10 m Detectors	−2.48	0.56	−3.57	−1.39	<0.001
Lure OFF After Trtmt:10 m Detectors	−0.08	0.49	−1.04	0.89	0.878
Lure ON:Center Detector	−4.26	1.22	−6.64	−1.88	<0.001
Lure OFF After Trtmt:Center Detector	0.10	0.56	−1.01	1.21	0.859
Preplanned Contrast	β	SE	LCL	UCL	*p* Value
Before Treatment, Lure OFF at Center Detector versus Lure ON at Center Detector	−3.55	1.17	−6.48	−0.63	0.005
Before Treatment, Lure OFF at Center Detector versus After Treatment, Lure OFF at Center Detector	0.83	0.51	−0.45	2.11	0.105
Lure ON at Center and 10 m Detectors versus Lure ON at 100 m Detector	3.53	0.63	1.97	5.10	<0.001
Lure ON at Center Detector versus Lure ON at 10 m Detector	−2.27	1.18	−5.20	0.67	0.072
Post hoc Contrast	β	SE	LCL	UCL	*p* Value
Before Treatment at 100 m Detectors versus Lure On at 100 m Detectors	0.71	0.35	0.03	1.38	0.041

**Table A4:** (top) Model AIC ranking table (top), model output for the selected top generalized linear mixed model (middle), and preplanned orthogonal contrasts (bottom) for the evening bat (*Nycticeius humeralis*) showing the response of received evening bat echolocation passes to the UV lure treatments, detector position within a site, environmental variables, and the linear (yday), quadratic (yday^2^), and cubic (yday^3^) day of the year at Prince William Forest Park and Marine Corps Base Quantico, VA, USA 2020–2021. Environmental variables included proportion of the night with measurable precipitation (Prop. Precip), average nightly temperature (Avg. Temp), average nightly windspeed (Avg. Windspeed), and the fraction of the moon illuminated at midnight (Frac. Moon Illum.). All numeric variables are centered and scaled. The coefficient values (β), standard errors (SE), 95% lower (LCL) and upper (UCL) confidence levels, and *p* values are shown. The contrasts test preplanned comparisons between lure treatment levels and bat detector positions within a site.

Model	AICc		ΔAIC		ω_i_
Global no Interaction	430.48		0.00		0.37
Distance and Weather	430.91		0.43		0.30
Distance Only	431.35		0.87		0.24
Lure and Distance	433.99		3.51		0.06
Global with Interaction	438.96		8.48		0.01
Lure Only	439.04		8.56		0.01
Weather Only	439.45		8.97		<0.01
Lure, Distance, and Interaction	440.87		10.39		<0.01
Lure and Weather	441.75		11.27		<0.01
Null Model	464.71		34.23		<0.01
Variables	β	SE	LCL	UCL	*p* Value
Lure ON	1.11	0.52	0.10	2.12	0.031
Lure OFF After Trtmt	1.62	0.76	0.12	3.12	0.034
10 m Detectors	−1.04	0.24	−1.51	−0.57	<0.001
Center Detector	0.32	0.23	−0.13	0.77	0.167
Prop. Precip	0.56	0.22	0.14	0.99	0.010
Avg. Temp	0.68	0.30	0.08	1.27	0.026
Avg. Windspeed	0.55	0.23	0.09	1.01	0.018
Frac. Moon Illum.	−1.44	0.78	−2.98	0.09	0.065
yday	67.26	34.68	−0.71	135.23	0.052
yday^2^	−37.85	20.58	−78.17	2.48	0.066
yday^3^	−9.51	19.43	−47.59	28.56	0.624
Contrast	β	SE	LCL	UCL	*p* Value
Before Treatment, Lure OFF at Center Detector versus Lure ON at Center Detector	1.11	0.52	−0.18	2.40	0.045
Before Treatment, Lure OFF at Center Detector versus After Treatment, Lure OFF at Center Detector	1.62	0.76	−0.29	3.53	0.045
Lure ON at Center and 10 m Detectors versus Lure ON at 100 m Detector	0.36	0.20	−0.13	0.85	0.068
Lure ON at Center Detector versus Lure ON at 10 m Detector	1.35	0.25	0.72	1.98	<0.001
