# Correction: Freeze et al. The Bat Signal: An Ultraviolet Light Lure to Increase Acoustic Detection of Bats. *Animals* 2025, *15*, 2458

**DOI:** 10.3390/ani16060868

**Published:** 2026-03-11

**Authors:** Samuel R. Freeze, Sabrina M. Deeley, Amber S. Litterer, J. Mark Freeze, W. Mark Ford

**Affiliations:** 1Department of Fish and Wildlife Conservation, Virginia Polytechnic Institute and State University, Blacksburg, VA 24061, USA; amberlitterer@vt.edu; 2Conservation Planning Assistance, Chesapeake Bay Ecological Services Field Office, U.S. Fish and Wildlife Service, Annapolis, MD 21401, USA; sabrina_deeley@fws.gov; 3Independent Electrical Engineer, Raleigh, NC 27613, USA; jmfreeze2@gmail.com; 4Virginia Cooperative Fish and Wildlife Research Unit, U.S. Geological Survey, Blacksburg, VA 24061, USA; wmford@vt.edu

## Table Legend

In the original publication [1], there was a mistake in the legend for Table 2. The legend points readers to the Supplemental Material to view full AICc model selection tables, when it should point readers to Table 3 and Appendix A. The correct legend appears below.
**Table 2.** Top model selected for each species of bat (top), all species combined (middle), and feeding buzzes (bottom) using Akaike Information Criteria, corrected for small sample size (AICc) at Prince William Forest Park and Marine Corps Base Quantico, VA, USA, 2020–2021. We considered models that were within two delta AICc of the top model and then selected the most parsimonious model that included the lure treatment effect. Models that did not converge were not included in the final model set. For full model selection tables, see Table 3 and Appendix A. Models that included a significant effect of lure treatment (*p* < 0.05) are indicated by an asterisk.

## Citation Mismatch

During the review process, Burnham and Anderson 2002 [47] was accidentally deleted and Robbins et al. 2008 [68] was accidentally merged with the citation preceding it. Additionally, Freeze and Ford 2021 was accidentally reinserted into the manuscript after being removed during the review process. These errors caused a mismatch between in-text citation reference numbers and their correct citation in the References section starting at citation number forty in the References section. The Freeze and Ford 2021 citation has been removed from References. The Burnham and Anderson 2002 [47] and Robbins et al. 2008 [68] citations have now been inserted in References. The corrected References [40–47] should read as follows:40.Agranat, I. *Detecting Bats with Ultrasonic Microphones: Understanding the Effects of Microphone Variance and Placement on Detection Rates*; Wildlife Acoustics, Inc.: Maynard, MA, USA, 2014; pp. 1–14.41.Ford, W.M. *Kaleidoscope 5.4.0beta0 Test on Expanded Data*; United States Department of The Interior, U.S. Geological Survey, Interior Ecosystems Division: Blacksburg, VA, USA, 2020; pp. 1–5.42.U.S. Fish and Wildlife Service. *Range-Wide Indiana Bat and Northern Long-Eared Bat Survey Guidelines*; U.S. Fish and Wildlife Service, Region 3: Bloomington, MN, USA, 2024; p. 95.43.Jameson, J.W. Buzzfindr: Automating the Detection of Feeding Buzzes in Bat Echolocation Recordings. *PLoS ONE*
**2024**, *19*, e0306063. https://doi.org/10.1371/journal.pone.0306063.44.Gorman, K.M.; Barr, E.L.; Ries, L.; Nocera, T.; Ford, W.M. Bat Activity Patterns Relative to Temporal and Weather Effects in a Temperate Coastal Environment. *Glob. Ecol. Conserv.*
**2021**, *30*, e01769. https://doi.org/10.1016/j.gecco.2021.e01769.45.Iowa Environmental Mesonet ASOS-AWOS-METAR Data Download. Available online: https://mesonet.agron.iastate.edu/request/download.phtml (accessed on 1 December 2020).46.U.S. Naval Observatory. Astronomical Applications Department. Fraction of the Moon Illuminated. Available online: https://aa.usno.navy.mil/data/MoonFraction (accessed on 8 May 2021).47.Burnham, K.P.; Anderson, D.R. *Model Selection and Multimodel Inference: A Practical Information-Theoretic Approach*; Springer: New York, NY, USA, 2002; ISBN 978-0-387-95364-9.

## Text Corrections

There was an error in the original publication [1]. In Section 2, Materials and Methods, Subsection 2.3, Field Methods, a sentence reads, “Although minimum distances between acoustic recorders to minimize spatial autocorrelation in real time are limited, 30 m was believed sufficient [40].” This sentence should read, “Although research into minimum distances between acoustic recorders to minimize spatial autocorrelation in real time is limited, 30 m was believed sufficient [40].”

A correction has been made to Section 2, Materials and Methods, Subsection 2.3, Field Methods, paragraph number one:

We conducted UV light trials during the summers of 2020 and 2021 from approximately mid-May to early August to coincide with the summer bat survey season [10]. We placed UV lights along single-track gravel or dirt roads and streams that served as bat flight corridors at PRWI and MCBQ (Figure 4). Survey sites were spaced so that no detector was within 1000 m of another within a given year to reasonably ensure spatial independence during operation. Although research into minimum distances between acoustic recorders to minimize spatial autocorrelation in real time is limited, 30 m was believed sufficient [40]. We chose to be conservative with our minimum distance since we were using a lure device that had potential to attract bats from a larger area. During the summer of 2020, fourteen sites were sampled, and eight sites were sampled during the summer of 2021, with six of those being resampled sites. The UV lure was deployed along the side of the corridor flyway (trail or wooded secondary road) so that there was ample open airspace around the lure for bats to easily forage. We placed Songmeter Mini Bat detectors (Wildlife Acoustics, Maynard, MA, USA) on approximately 2 m tall poles (Mr. LongArm Inc., Greenwood, MO, USA) along the same side of the flyway as the UV lure. One detector was placed at the UV lure and aimed directly at the lights during the summer of 2020, with two during the summer of 2021 to provide redundancy. The horizontal distance between the center detector and UV lure varied slightly between sites but was typically about 1–2 m. To understand the potential effects of distance on UV light placement, we also placed detectors at 10 m and 100 m from the light, with the latter serving as our in-site control (Figure 5).

Additionally, In Section 5, Conclusions, a sentence reads, “However, the response is species-dependent, with generally edge-foraging generally showing the greatest response, and one species possibly being deterred from the illuminated area”. This sentence should read, “However, the response is species-dependent, with generally edge-foraging species showing the greatest response, and one species possibly being deterred from the illuminated area.”

A correction has been made to Section 5, Conclusions, paragraph number one:

Our study was one of the first comprehensive attempts to use a UV light lure to increase the acoustic detection of bats. We demonstrated that UV lures show promise by increasing echolocation and feeding activity, including that of a federally endangered species. However, the response is species-dependent, with generally edge-foraging species showing the greatest response, and one species possibly being deterred from the illuminated area. More research is warranted with the hope of eventually using UV lures to reduce the time and effort required to survey for rare bat species. The results also corroborate similar efforts to use UV lights to improve foraging opportunities for bats impacted by White-Nose Syndrome as they enter hibernation in the fall and emerge in the spring [17].

## References

There was an error in reference 10, which should appear as below.

10.U.S. Fish and Wildlife Service. Range-Wide Indiana Bat Summer Survey Guidance. Available online: http://www.fws.gov/midwest/Endangered/mammals/inba/inbasummersurveyguidance.html (accessed on 13 September 2015).

The authors state that the scientific conclusions are unaffected. This correction was approved by the Academic Editor. The original publication has also been updated.
